# Evaluating the impact of a standardised intervention for announcing decisions of withholding and withdrawing life-sustaining treatments on the stress of relatives in emergency departments (DISCUSS): protocol for a stepped-wedge randomised controlled trial

**DOI:** 10.1136/bmjopen-2024-087444

**Published:** 2024-09-05

**Authors:** Anne Termoz, Fabien Subtil, Pauline Drouin, Mathilde Marchal, Manon Verroul, Carole Langlois, Estelle Bravant, Laurent Jacquin, Bénédicte Clément, Damien Viglino, Daniel Roux-Boniface, Frédéric Verbois, Marine Demarquet, Xavier Dubucs, Delphine Douillet, Karim Tazarourte, Anne-Marie Schott-Pethelaz, Julie Haesebaert, Marion Douplat

**Affiliations:** 1 Research on Healthcare Performance (RESHAPE), INSERM U1290, Université Claude Bernard Lyon 1—Domaine de Rockefeller, Lyon, Rhône-Alpes, France; 2 Pôle Santé Publique, Service Recherche et Epidémiologie Cliniques, Hospices Civils de Lyon, Lyon, Auvergne-Rhône-Alpes, France; 3 Pôle Santé Publique, Service de Biostatistique et Bio-informatique, Hospices Civils de Lyon, Lyon, Auvergne-Rhône-Alpes, France; 4 CNRS, UMR 5558, Laboratoire de Biométrie et Biologie Évolutive, Université Claude Bernard Lyon 1, Villeurbanne, Auvergne-Rhône-Alpes, France; 5 Hôpital Edouard Herriot, Service d’Accueil des Urgences, Hospices Civils de Lyon, Lyon, Auvergne-Rhône-Alpes, France; 6 Hôpital de la Croix-Rousse, Service d’Accueil des Urgences, Hospices Civils de Lyon, Lyon, Auvergne-Rhône-Alpes, France; 7 Hôpital Nord, Service d’Accueil des Urgences, Centre Hospitalier Universitaire Grenoble Alpes, Grenoble, France; 8 Hôpital Gabriel Montpied, Service d’Accueil des Urgences, Centre Hospitalier Universitaire de Clermont-Ferrand, Clermont-Ferrand, France; 9 Centre Hospitalier Nord Ouest, Service d’Accueil des Urgences, Hopital de Villefranche-sur-Saone, Villefranche-sur-Saone, Auvergne-Rhône-Alpes, France; 10 Centre Hospitalier Fleyriat, Service d’Accueil des Urgences, Centre Hospitalier de Bourg-en-Bresse, Bourg-en-Bresse, Rhône-Alpes, France; 11 Hôpital Larrey, Service d’Accueil des Urgences, Centre Hospitalier Universitaire de Toulouse, Toulouse, Occitanie, France; 12 Hôpital Larrey, Service d’Accueil des Urgences, Centre Hospitalier Universitaire d'Angers, Angers, Pays de la Loire, France; 13 Hôpital Lyon Sud, Service d’Accueil des Urgences, Hospices Civils de Lyon, Lyon, Auvergne-Rhône-Alpes, France

**Keywords:** stress, psychological, emergency departments, decision making, patient-centered care, randomized controlled trial

## Abstract

**Introduction:**

The decisions of withholding or withdrawing life-sustaining treatments are difficult to make in the context of emergency departments (EDs) because most patients are unable to communicate. Relatives are thus asked to participate in the decision‐making process, although they are unprepared to face such situations. We therefore aimed to develop a standardised intervention for announcing decisions of withholding or withdrawing life-sustaining treatments in EDs and assess the efficacy of the intervention on the stress of relatives.

**Methods and analysis:**

The DISCUSS trial is a multicentre stepped-wedge cluster randomised study and will be conducted at nine EDs in France. A standardised intervention based on human simulation will be codesigned with partner families and implemented at three levels: the relatives, the healthcare professionals (HCP) and the EDs. The intervention will be compared with a control based on treatment as usual. A total of 538 families are planned to be included: 269 in the intervention group and 269 in the control group. The primary endpoint will be the symptoms of post-traumatic stress disorder (PTSD) at 90 days. The secondary endpoints will be symptoms of PTSD at 7 and 30 days, diagnosis of PTSD at 90 days and anxiety and depression scores at 7, 30 and 90 days. Satisfaction regarding the training, the assertiveness in communication and real-life stress of HCPs will be measured at 90 days.

**Ethics and dissemination:**

This study was approved by the ethics committee Est III from Nancy and the French national data protection authority. All relatives and HCPs will be informed regarding the study objectives and data confidentiality. Written informed consent will be obtained from participants, as required by French law for this study type. The results from this study will be disseminated at conferences and in a peer-reviewed journal.

**Trial registration number:**

NCT06071078.

**STRENGTHS AND LIMITATIONS OF THIS STUDY**

- The strategy proposed is based on a standardised intervention that will be implemented at three levels (healthcare professionals (HCP), relatives and the emergency department (ED)) and will be codesigned with partner families.
- The originality of the proposed protocol is the involvement of partner families, which has never been tested and the use of the Theoretical Domain Framework will enhance the expected efficacy of the training.
- A stepped-wedge randomised controlled trial provides a high level of evidence while adopting the pragmatic approach needed to study the implementation of training in practice.
- Another strength of this study is the use of a mixed methodology, including a qualitative component, to study the experiences of HCP and families in greater depth.
- The main limitation of the intervention will be the reproducibility and the adherence to the training in all EDs.

## Introduction

### Context of emergency departments (EDs)

 Death in the ED occurs in 0.3% to 0.5% of emergency admissions, representing approximately 26 000 deaths per year in France.^1^ For 80% of these deceased patients, a decision of withholding or withdrawing life-sustaining treatments was made in the ED.^1 2^ These decisions are complex in the context of EDs because data regarding patients’ medical history and previous functional limitations are often lacking. Moreover, most patients are unable to communicate or make decisions due to their clinical state and advance directives are only very rarely available. Nevertheless, ethical aspects must be respected and families are thus asked to participate in the decision‐making process concerning the withdrawal or withholding of life-sustaining treatments, although they are unprepared to face such situations. In addition, the short delay between ED admission and the decisions increases the stress and anxiety of families who are unprepared for the announcement. The lack of time, the inappropriate places to make announcements in the EDs and the lack of training also worsen the situation and add difficulty for healthcare professionals (HCP).^3^ Physicians facing these situations report a feeling of loneliness and being overwhelmed^3^ and both nurses and physicians report difficulties in communicating with relatives regarding such decisions.^4^

### Impact on relatives

The impact on relatives of the announcement of a decision to withdraw or withhold life-sustaining treatments has been widely explored in the context of intensive care units (ICU).^5 6^ In this setting, it has been shown that symptoms of anxiety and depression are correlated with the onset of post-traumatic stress disorder (PTSD), the symptoms of which were more important among the relatives of deceased patients having undergone a decision of withholding or withdrawing life-sustaining treatments and when the relatives were involved in the decision-making process.^7^ The impact on relatives in the context of EDs, however, has rarely been studied. In a previous study, we found that relatives displayed symptoms of anxiety and depression after the announcement, symptoms which persisted over time.^8^

### Strategies for reducing the impact on relatives

With the aim of preventing the onset of PTSD symptoms after death or a decision of withdrawing or withholding life-sustaining treatments, several studies have evaluated communication strategies in the ICU setting.^9^,^12^ For example, it has been shown that the training of nursing staff in communication skills or the use of a brochure for dealing with the families of patients who have died in the ICU reduces the incidence of PTSD symptoms among relatives.^9^ Human simulation is a pedagogical technique for learning interpersonal skills through role playing that has shown its effectiveness not only in communication training but also in developing empathy compared with the standardised patient method.^13^ The use of simulation has been demonstrated to teach end-of-life communication among health students^14^ and among HCP, with a positive impact on the families in the ICU.^15^

We therefore aimed to develop a standardised intervention using human simulation for announcing decisions of withholding or withdrawing life-sustaining treatments in EDs. We present here the protocol for the DISCUSS trial, a multicentre stepped-wedge cluster randomised trial evaluating the impact of a standardised intervention for announcing decisions of withholding or withdrawing life-sustaining treatments in EDs on the stress of relatives. Partner families will be involved in the design of the intervention, which will then be implemented at three levels: the HCP, the relatives and the ED. Involving patients and families as partners in research has been shown to improve the social acceptability of studies and facilitate knowledge transmission strategies among patients and HCP.^16^ Moreover, recommendations to promote patient involvement in emergency medicine research have recently been published.^16^ The group of Wright *et al* have also adapted the framework for patient partnership in research to the contextual challenges of the EDs for each stage of the research process, thus facilitating patient engagement. This practice, however, is not yet largely implemented in France.^17 18^

### Objectives

The main objective of the trial is to compare PTSD symptoms presented by families 90 days after an announcement of a decision to withhold or withdraw life-sustaining treatments in EDs between announcements made following the implementation of the standardised intervention and those made according to usual practices.

The secondary objectives are to study the efficacy of the standardised intervention on the following patient-reported outcomes: PTSD, anxiety, depression, families’ experiences of the announcement and impact on the socioprofessional life of family members during 90 days. We will also measure the efficacy of the standardised intervention on HCP satisfaction regarding the training, assertiveness in communication, self-confidence, real-life stress levels in the work environment, behavioural changes and the experience of the announcement from the HCP perspective. A process evaluation of the intervention implementation will be conducted using a mixed-method approach based on the Medical Research Council guidance.^19 20^ Semistructured interviews will be conducted to understand the barriers and facilitators perceived by the relatives and HCP.

## Methods and analysis

### Study design

**Figure 1:**
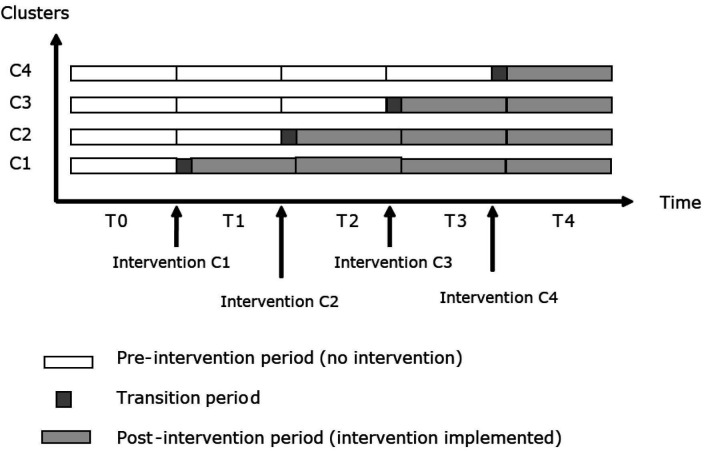
Study design and clusters randomization according to the different time periods.

The DISCUSS trial is a multicentre stepped-wedge cluster randomised study, which will be conducted at nine EDs in France. The nine EDs will be arranged into four groups, each group thus being composed of two to three EDs. The trial will be performed in five steps, and each group of clusters will be randomly assigned to one step. After a preintervention period (control), the intervention will be sequentially implemented in the groups at different times, according to a random allocation sequence generated by computer by the biostatistical team in charge of data analysis and blinded to group composition. All clusters will eventually receive the intervention (figure 1).

The control group will be composed of relatives included in the preintervention period, and the intervention group will be composed of relatives included after the implementation of the intervention. No relative will be included during the transition periods corresponding to a 1-month period during which the intervention will be deployed in the ED.

For the qualitative analysis, semistructured interviews will be conducted with relatives: between 15 and 20 interviews will be planned depending on data saturation. This number is planned based on the available literature on data saturation.^21 22^ However, if new information emerges from the final interviews, additional interviews will be planned.

The reporting of the study protocol follows the Standard Protocol Items: Recommendations for Interventional Trials guidelines,^23^ and the description of the planned intervention is guided by the template for intervention description and replication checklist.^24^ Patient and public involvement is reported according to the GRIPP V.2 reporting checklist.^25^

### Setting and patients

The target population of relatives will be composed of designated trusted persons, family members or friends to whom the first announcement of a decision to withhold or withdraw life-sustaining treatments is made by a physician participating in the study. The study will include only adult patients and adult relatives. For each patient, one relative will be included. Another emergency physician who does not make the announcement will inform the patients and their relatives and obtain their consent before their inclusion in the study. Relatives for whom the announcement is made entirely by telephone will not be included.

### Design of a standardised three-level intervention

During the first phase of the protocol, the detailed content of the intervention will be codesigned with partner families who have previously experienced an announcement of a decision of withholding or withdrawing life-sustaining treatments in the ED. Semistructured interviews with the partner families and two workshops will be conducted by professionals trained in qualitative research to develop and validate the intervention. The intervention will also be designed according to national and international recommendations^23^,^25^ and using the Theoretical Domain Framework (TDF),^26 27^ specifically guided by the following dimensions: knowledge, skills, social/professional role and identity, beliefs about capabilities, optimism, intentions, environmental context and emotion.

### Intervention implementation

The intervention will then be deployed at three levels. At the level of the HCP (physicians and nurses), the intervention will consist in a two-step human simulation training approach. First, two pairs of HCP (one physician and one nurse) will be trained in a simulation centre by a trainer specialised in simulation concerning decisions of withholding or withdrawing life-sustaining treatments. The trained HCP will then implement the training in situ in the ED with the help of the specialised trainer. The scenarios used in the human simulation training will be constructed during the first phase with the partner families based on their experiences. The intervention will also include video testimonials from partner families and a training manual including all the objectives and skills targeted by the training, which will be made available electronically to all HCP. At the level of the relatives, an information booklet on the decision-making process for withdrawing or withholding life-sustaining treatments and end-of-life care in the context of EDs, codeveloped with the partner families, will be given to the relatives included in the study. Finally, at the level of the ED, changes will be made to provide a dedicated room for the announcement. Time will be allocated specifically for making the announcement, which will be made by a team of HCP (a physician and a nurse).

### Control (treatment as usual)

The control group will be composed of relatives who have received an announcement of a decision of withholding or withdrawing life-sustaining treatments from an HCP who has not undergone the standardised intervention, that is, before the deployment of the training in the ED.

A survey of the usual practices for the announcement of decisions of withholding or withdrawing life-sustaining treatments in each centre will be carried out before the beginning of the intervention. More specifically, information regarding possible training received by the HCP on the subject, existence of an announcement procedure and/or a place dedicated to the announcement in the ED, the presence of a psychologist intervening during the announcements and the possible recourse to religious representatives will be collected.

### Measurement of patient outcomes

The study schedule reporting the time points for data collection is presented in online supplemental table 1.

### Primary outcome

Symptoms of PTSD at 90 days will be measured by the Impact of Events Scale (IES),^28^ a 15-item self-administered questionnaire validated in French,^29^ which measures symptoms of intrusion, avoidance and hyperarousal experienced over the past 7 days. Each item is graded from 0 to 4 and the final score ranges from 0 to 75. Higher scores reflect the greater presence of symptoms, and a score above 30 indicates the presence of symptoms suggestive of PTSD requiring referral for treatment. For all the subjects included, the questionnaire will be carried out over the phone, by a psychologist from the coordinating centre blinded to group allocation, 90 days after the announcement in the ED.

### Secondary outcomes

Symptoms of PTSD at 7 and 30 days will be measured by the IES. PTSD will be measured at 90 days using the Post-traumatic Stress Disorder Checklist for Diagnostic and Statistical Manual of Mental Disorders (DSM-5).^30 31^ Anxiety and depression scores will be measured at 7, 30, and 90 days by the Hospital Anxiety and Depression Scale (HADS), which has been validated in French.^32^,^34^ The relatives’ experiences of the announcement will be collected at 7 days through a questionnaire investigating feelings and experience. The presence of at least one work absenteeism related to the ED visit as well as the number of days of work absenteeism will be assessed within 90 days of the announcement.

### Measurement of HCP outcomes

Satisfaction with the training received will be evaluated by an ad-hoc questionnaire 90 days after training. Assertiveness in communication will be measured at baseline and at 90 days using the Cungi and Rey scale.^35^ Self-confidence will be assessed at baseline and at 90 days by an ad hoc questionnaire. Real-life stress levels in the work environment will be evaluated at baseline and at 90 days using the Karasek scale.^36 37^ Behavioural changes will be collected after training by an ad hoc questionnaire, and experience of the announcement will be collected by individual semistructured interviews 90 days after the training.

### Implementation process evaluation

Elements that are independent of the intervention that may have modified the implementation or effect of the intervention will be studied using quantitative and qualitative approaches. Regarding training delivery, the number and profile of HCP trained in the simulation centre and in situ will be collected. Regarding protocol implementation, the fidelity of the intervention deployed and the adaptations made will be recorded. The participation and satisfaction of HCP regarding the training will be collected. The proportion of announcements during which strong emotional reactions or conflicts/complaints arised as well as the unexpected effects of using the announcement protocol on the relatives, HCP and EDs will be collected by questionnaire and during semistructured interviews. The organisational factors in the EDs that can influence the deployment of the intervention will be researched.

### Data collection

Inclusion began in January 2024 and will end in September 2025, with the last patients included being monitored until the end of December 2025. Data will be collected from the ED medical charts by research assistants. For each relative included in the trial, a case report form will be completed with his/her characteristics (age, sex) and the characteristics of the patient.

Relatives will be contacted at 7, 30 and 90 days to collect outcomes. This will be done by telephone by a psychologist of the study coordinating centre. The data collected are listed in online supplemental table 1.

### Data management

All information required by the protocol will be recorded in an electronic case report form (eCRF). This eCRF, specific to the study, will be developed by a data manager from the Hospices Civils de Lyon on the Ennov Clinical software (V.8.2.50).

The data set will be computerised in a coded way, in accordance with the law for data protection and freedom of information (Article L.1121-3 of the French Public Health Code). The study families will be identified by a unique study inclusion number and by the first initial of their surname and of their given name.

Data will be entered, as soon as they are collected, by the authorised persons (investigator and personnel recorded on the delegation log) according to the law for data protection and freedom of information.

Access to the data will be restricted to the authorised persons only. Authentication will be made using passwords, which will be regularly changed. The investigators and clinical research assistants of an investigating centre will only have access to the data for their patients and will enter the data directly into the eCRF using a secured website. The investigator will be responsible for the reliability of the data entered. Throughout the length of the study, the data will be stored in an ISO 27001-certified data centre and backed up daily.

### Sample size

A mean IES of 33 is expected at 90 days during the control periods (SD of 9) and a relative reduction of 12% of the mean score is expected with the intervention. Under thess hypotheses, for a 90% power and a 5% two-sided alpha level, 216 families would be necessary. By considering an intraclass correlation coefficient of 0.35, 9 EDs divided into 4 groups and 15% of loss to follow-up, a total of 538 families will be necessary.

### Recruitment

The nine participating centres were chosen for their high inclusion potential with over 300 000 annual ED admissions and approximately 600 decisions of withholding or withdrawing life-sustaining treatments per year. In a previous study, a mean of six patients were included per month in two of the participating centres.^38^ Based on this experience and given the similar activity of the other investigating centres, recruiting three families per month and per centre seems feasible.

### Statistical analysis

The IES Score will be analysed by a linear mixed effect model. It will consider as fixed effect, the intervention (period with or without the intervention), the measurement time point (7, 30 and 90 days), and an interaction between time point and period. The model will also be adjusted on the following factors:

1. Regarding the patient: age, institutionalised patient, advance directives, death of the patient during the 90 days.
2. Regarding the relatives: age, sex, relationship with the patient, lifestyle (single or in a relationship), frequency of contact with the patient over the past 6 months, history of psychological or psychiatric care, religious beliefs.
3. Regarding the ED at the time of the announcement: work overload, doctor/nurse/patient ratio in the ED at the time of the announcement.

The model will include as random effect, a random intercept by ED and a random intercept and slope by family nested in the ED level. The effect of the intervention at 90 days will be recalculated from the coefficients of the model; it will be quantified by the difference of mean with the associated 95% CI and tested by a Wald test. A period effect will be tested in the model. A secondary analysis for the primary endpoint will be performed by considering IES as a binary variable, a value above 30 indicating a high risk of PTSD. The analysis will be similar to the one previously described, but performed using a mixed effect logistic regression model.

The analysis of PTSD at 7 and 30 days will be performed by calculating the effect of the intervention from the coefficients of the aforementioned model.

The analysis of anxiety and depression will be performed similarly to one of the primary endpoint and will focus specifically on the effect of the intervention at day 7 and on the effect of the intervention on the slope. A binary analysis will also be performed, by considering anxiety or depression when the HADS scores are above 8.

The analysis of PTSD symptoms at 90 days will be performed by a mixed effect logistic regression model, considering only the ED as random intercept. The comparison of the proportion of relatives with at least one work absenteeism will be performed by a mixed effect logistic regression model. The percentages of relatives for whom the announcement was considered smooth will be compared. A threshold of 0.05 will be considered significant. Analyses will be performed using R and SAS software (version 9.4).

### Qualitative study

During the study, a psychologist will propose to a sample of relatives from the intervention group to participate in individual semistructured interviews. These interviews will help better identify their perceptions about the announcement made, communication and their involvement in the decision-making process. The interviews will take place by phone. They will be conducted by a psychologist and based on an interview guide elaborated and validated by the steering committee. Similarly, interviews of the HCP will be carried out by a psychologist to identify the perceptions concerning the proposed training, the changes in practices linked to the intervention and its components (guided by TDF dimensions), the facilitators and barriers regarding the implementation of the intervention and the different components of the intervention and their perception concerning the participation of partner families in the intervention.

All the interviews will be audio-recorded and transcribed for analysis. The analysis will focus on data from verbatim and interview notes. A thematic analysis of the content following the approach proposed by Bardin^39^ will be carried out using NVIVO software (Nvivo QSR International). A vertical and transversal analysis will be carried out to categorise the verbatim into themes and subthemes. The analysis grid will follow the themes of the interview grid, enriched during the analysis of emerging themes and subthemes. The different data sources (interviews and notes) and populations (families and HCP) will be triangulated. The results will then be combined with the results of the quantitative data and collected to analyse the implementation.

### End of follow-up

The follow-up of the relatives will end at 90 days after their inclusion. End-of-study data will be collected by telephone by a psychologist; the data collected is specified above.

For relatives participating in the qualitative study, participation in the interview will mark the end of the study. Given the nature of the intervention and the low risk of serious research-related adverse events, no independent data monitoring committee is planned for this study. Only the statistician and the coordinating centre will have access to the final trial dataset.

### Patient and public involvement

Families of patients who have experienced an announcement of a decision to withhold or withdraw life-sustaining treatments will be included as partner families to codesign the simulation scenario and make video testimonials dedicated to the HCP; these will be used in the human simulation training and during the in situ simulation. They will be recruited by phone among families who have visited the coordinating centre ED. We plan to include four family members. They will be offered to participate in two workshops and/or the making of video testimonials; their participation will thus concern the intervention codesign phase of the project.

## Ethics and dissemination

### Ethics approval and consent to participate

This study was approved by the ethics committee Est III from Nancy and the French national data protection authority. The study was registered in ClinicalTrials.gov under the reference NCT06071078. All relatives and HCP will be informed regarding the study objectives and data confidentiality. Written informed consent will be obtained from participants, as required by French law for this study type (online supplemental file).

### Dissemination

A scientific paper will be published in a peer-reviewed journal and a written communication will be sent to the study participants to present the main results.

Only the full protocol may be made available to the public on request to the corresponding author.

## Discussion

To our knowledge, this is the first multicentre stepped-wedge cluster randomised study with a high level of evidence focused on evaluating the impact of announcing decisions of withholding or withdrawing life-sustaining treatments on stress for the relatives in the ED setting. The strategy proposed is based on a standardised intervention which will be implemented at three levels (HCP, relatives and the ED) and which will be codesigned with partner families.

The originality of the proposed protocol is the involvement of partner families, which has never been tested, and the use of TDF, which will enhance the expected efficacy of the training. The involvement of partner families in research is only an emerging approach in EDs in France, despite the fact that adaptations of the conceptual frameworks to the context of EDs have been proposed.^17^ We have previously shown the benefits of such partnership for both HCP and patients in the EDs.^18^

The main limitation of the intervention will be the reproducibility and the adherence to the training in all EDs. This is why the training was designed using a two-level human simulation approach: first, two pairs of HCP per ED will be trained and these will in turn be helped to develop the training in situ. In addition, an implementation process evaluation will be conducted to determine whether the training was carried out effectively in the EDs, how many professionals were trained and to gain a better understanding of the contextual factors that helped or hindered the implementation. Another limitation is the risk of relatives being lost to follow-up due to the emotional context of the decision. The evaluation will thus be carried out by a psychologist with experience of these announcements. Finally, the turnover of the HCP in EDs could be a challenge for effectively implementing the intervention over time, which is why a training manual including all the objectives and skills targeted by the training and video testimonials of partner families will be developed.

The strengths of this study are that it uses a mixed methodology including a qualitative component to study the experiences of HCP and families in greater depth. In addition, the protocol uses a patient-centred research approach by including partner families in the first phase of the study. These partner families, having experienced an announcement of a decision to withhold and withdraw life-sustaining treatments, will be able to help develop and validate the training (booklet, scenario).

The chosen methodology is a stepped-wedge randomised controlled trial to assess the effectiveness of this complex intervention. This design provides a high level of evidence while adopting the pragmatic approach needed to study the implementation of training in practice. One of the strengths of this methodology is that it makes it possible to analyse the effect of time and to consider each ED as its own control, therefore taking into account the effect differences between clusters. In this case, the effect differences are potentially significant due to the differences in size and organisation of the participating EDs, making it the methodology of choice compared with a parallel cluster trial. In addition, this method will reduce the risk of contamination during the study, since each ED undergoes the training at the end of the study.

The proposed protocol may contribute to improve the announcement of decisions to withdraw or withhold life-sustaining treatments and reduce the impact of these announcements on relatives. It will also strengthen relational skills for HCP, improve their relationships with families and enhance patient and family-centred care. In case of positive results, the standardised intervention may be transferred to other EDs.

## supplementary material

10.1136/bmjopen-2024-087444 online supplemental file 1

10.1136/bmjopen-2024-087444 online supplemental file 2
