# Supplementary material for: Evaluating the impact of a standardised intervention for announcing decisions of withholding and withdrawing life-sustaining treatments on the stress of relatives in emergency departments (DISCUSS): protocol for a stepped-wedge randomised controlled trial
Source: BMJ Open. 2024 Sep 5;14(9):e087444. doi: 10.1136/bmjopen-2024-087444 (PMC11381699; doi:10.1136/bmjopen-2024-087444)
Supplement: online supplemental file 1 [file bmjopen-14-9-s001.pdf]

1

		Families			Healthcare professionals				Emergency Department	Instrument
**Timing**	Inclusion	7 days	30 days	90 days	Baseline	Before Training	After Training	90 days after training	After Training	
Baseline information	x									
**PRIMARY OUTCOME**										
Symptoms of post-traumatic stress				x						Impact of Events Scale
**SECONDARY OUTCOMES**										
Symptoms of post-traumatic stress		x	x							Impact of Events Scale
Posttraumatic stress disorder				x						PCL-5
Anxiety and depression		x	x	x						Hospital Anxiety and Depression scale
Families' experiences of the announcement		x								Reported by the families using a structured

Presence of at least one work absenteeism related to the ED visit	x						questionnaire
Families' experiences in the training group	x						Individual semi-structured interview
Satisfaction with the training					x		Ad-hoc questionnaire
Impact of partner families' involvement in training			x	x			Reported by HCP using a structured questionnaire
Assertiveness in communication		x			x		Cungi and Rey scale
Self-confidence		x			x		Ad-hoc questionnaire
Real-life stress levels in the work environment		x			x		Karasek scale
Behavioural changes				x			Ad-hoc questionnaire
Experience of the announcement					x		Individual semi-structured interview
Implementation of the protocol in practice						x	Ad-hoc questionnaire and semi-structured interview

Participation and satisfaction of professionals with the training			x	Ad-hoc questionnaire and semi-structured interview
Reactions of families			x	Ad-hoc questionnaire and semi-structured interview
Unexpected effects of the use of the announcement protocol			x	Ad-hoc questionnaire and semi-structured interview
Favourable or limiting contextual factors – departmental organizational factors			x	Ad-hoc questionnaire and semi-structured interview
Favourable or limiting contextual factors – leadership facilitator in the team			x	Ad-hoc questionnaire and semi-structured interview
Favourable or limiting contextual factors – number of emergency department visits with flow analysis			x	Ad-hoc questionnaire and semi-structured interview
Favourable or limiting contextual factors –			x	Ad-hoc questionnaire and semi-structured

characteristics of the patients cared of					interview

PCL-5: Posttraumatic Stress Disorder Checklist for DSM-5, HCP: healthcare professionals

Table 1: Study schedule
