# Supplementary material for: Evaluating the impact of a standardised intervention for announcing decisions of withholding and withdrawing life-sustaining treatments on the stress of relatives in emergency departments (DISCUSS): protocol for a stepped-wedge randomised controlled trial
Source: BMJ Open. 2024 Sep 5;14(9):e087444. doi: 10.1136/bmjopen-2024-087444 (PMC11381699; doi:10.1136/bmjopen-2024-087444)
Supplement: online supplemental file 2 [file bmjopen-14-9-s002.pdf]

HCL HOSPICES CIVILS DE LYON — LA RECHERCHE	**FORMULAIRE DE CONSENTEMENT ETUDE [PRINCIPALE]**

Impact d'un protocole d'annonce des limitations et arrêts des thérapeutiques aux urgences sur le stress des familles

Etude DISCUSS

Version 1 du 11/05/2023

Je soussigné(e) [*Nom, Prénom*] .... consens librement à participer à cette recherche telle que décrite dans la lettre d'information et je confirme les points suivants :

- J'ai eu le temps de lire ces informations, de réfléchir à l'étude et j'ai obtenu des réponses appropriées à mes questions.
- J'ai bien été informé(e) de la nature des objectifs de la recherche, des risques potentiels et des contraintes liées à cette recherche.
- Je certifie être affilié(e) à un régime de sécurité sociale ou bénéficiaire d'un tel régime, sauf dérogation exceptionnelle.
- J'ai le droit de refuser de participer à la recherche ou de retirer mon consentement à tout moment sans conséquence sur ma prise en charge médicale et sans encourir aucune responsabilité ni préjudice de ce fait.
- J'ai bien compris la possibilité qui m'est réservée d'interrompre ma participation à cette recherche à tout moment sans avoir à justifier ma décision et j'informerai l'investigateur qui me suit dans la recherche. Cela ne remettra pas en cause la qualité des soins ultérieurs.
- J'ai bien compris que l'investigateur peut interrompre à tout moment ma participation à la recherche s'il le juge nécessaire.
- J'ai bien noté que je dispose d'un droit d'accès, de rectification, de limitation et, le cas échéant, d'opposition et d'effacement, concernant le traitement de mes données personnelles. Ces droits s'exercent en premier lieu auprès de l'investigateur qui me suit dans le cadre de cette recherche et qui connaît mon identité.
- J'ai bien pris connaissance que cette recherche a reçu l'avis favorable du Comité de Protection des Personnes Est III. Le promoteur de la recherche a souscrit une assurance de responsabilité civile en cas de préjudice auprès de la Société Relyens Mutual Insurance, 18 rue Edouard Rochet, 69008 Lyon.
- Mon consentement ne décharge en rien l'investigateur et le promoteur de la recherche de leurs responsabilités à mon égard. Je conserve tous mes droits garantis par la loi.
- Les résultats globaux de la recherche me seront communiqués à la fin de la recherche, si j'en fais la demande auprès de l'investigateur.
- Après le commencement de la recherche, je pourrais à tout moment demander des informations complémentaires au professionnel de santé recueillant mon consentement

- Deux exemplaires originaux de ce formulaire de consentement ont été établis : un m'a été remis, le second gardé par l'investigateur. Ils seront conservés dans le dossier de l'étude au minimum 15 ans après la fin de la recherche.
- J'ai été informé(e) sur la façon dont mes données personnelles, utilisés et partagés comme décrit dans ce document.

J'accepte que mes données personnelles codées soient utilisés pour d'autres recherches liées à la santé ou à la médecine, exclusivement à des fins scientifiques dans le domaine d'annonce des décisions de limitation ou arrêt des thérapeutiques sachant que je peux à tout moment retirer mon accord.	Oui ☐ Non ☐

Prénom et Nom du/de la participant(e) en majuscules [date de naissance pour éviter les homonymies] ……………………………………………………………………………………………|___|___| / |___|___| / |___|___|___|___|
Date de signature |___|___| / |___|___| / |___|___|___|___|
Signature du/de la participant(e)

Prénom et nom de l'investigateur ou du médecin / professionnel de santé qui le représente ayant informé le/la participant(e) ……
Date de signature |___|___| / |___|___| / |___|___|___|___|
Signature de l'investigateur ou du médecin / professionnel de santé qui le représente ayant informé le/la participant(e)
