# Supplementary material for: Evaluating the impact of a standardised intervention for announcing decisions of withholding and withdrawing life-sustaining treatments on the stress of relatives in emergency departments (DISCUSS): protocol for a stepped-wedge randomised controlled trial
Source: BMJ Open. 2024 Sep 5;14(9):e087444. doi: 10.1136/bmjopen-2024-087444 (PMC11381699; doi:10.1136/bmjopen-2024-087444)
Supplement: Supplementary file 3 [file bmjopen-2024-087444.reviewer_comments.pdf]

PEER REVIEW HISTORY

BMJ Open publishes all reviews undertaken for accepted manuscripts. Reviewers are asked to complete a checklist review form and are provided with free text boxes to elaborate on their assessment. These free text comments are reproduced below.

ARTICLE DETAILS

**TITLE (PROVISIONAL)**	Evaluating the impact of a standardised intervention for announcing decisions of withholding and withdrawing life-sustaining treatments on the stress of relatives in emergency departments (DISCUSS): protocol for a stepped-wedge randomised controlled trial
**AUTHORS**	Termoz, Anne; Subtil, Fabien; Drouin, Pauline; Marchal, Mathilde; Verroul, Manon; Langlois, Carole; Bravant, Estelle; Jacquin, Laurent; Clément, Bénédicte; Viglino, Damien; Roux-Boniface, Daniel; Verbois, Frédéric; Demarquet, Marine; Dubucs, Xavier; Douillet, Delphine; Tazarourte, Karim; Schott-pethelaz, Anne-Marie; Haesebaert, Julie; Douplat, Marion

VERSION 1 - REVIEW

**REVIEWER NAME**	Menon, Mahesh Radhakrishnan
**REVIEWER AFFILIATION**	High Bright Healthcare Private Limited, Pain and palliative care
**REVIEWER CONFLICT OF INTEREST**	None
**DATE REVIEW RETURNED**	19-Jun-2024

**GENERAL COMMENTS**	Page 4, Line 74: The secondary endpoint of PTSD at 90 days is repeated- this is the primary endpoint.<br><br>BMJ Open would require the name of the first 3 authors followed by et al where applicable.

**REVIEWER NAME**	Chocron, Richard
**REVIEWER AFFILIATION**	Paris Centre of Cardiovascular Research
**REVIEWER CONFLICT OF INTEREST**	none
**DATE REVIEW RETURNED**	10-Jul-2024

**GENERAL COMMENTS**	The protocol DISCUSS for a randomised trial evaluating the impact of a standardised intervention for announcing decisions of withholding and withdrawing life-sustaining treatments on the stress of relatives in emergency departments – This protocol is well presented with all informations needed to understand the purpose of the study. After carefull reading, I think this article can be accepted for publication.

VERSION 1 – AUTHOR RESPONSE

Page 4, Line 74: The secondary endpoint of PTSD at 90 days is repeated- this is the primary endpoint.

Authors’ response: The primary endpoint was symptoms of PTSD at 90 days assessed using the Impact of Event Scale while the secondary endpoint was diagnosis of PTSD at 90 days assessed using the Post Traumatic Stress Disorder Checklist for DSM-5. We have added ‘diagnosis of PTSD’ line 75 p 4 for more details.
